# The role of B cells in the pathogenesis of type 1 diabetes

**DOI:** 10.3389/fimmu.2024.1450366

**Published:** 2024-12-24

**Authors:** Ya-nan Wang, Ruihua Li, Yaxuan Huang, Hui Chen, Hao Nie, Lian Liu, Xiaoting Zou, Jixin Zhong, Bing Zheng, Quan Gong

**Affiliations:** 1 Department of Immunology, School of Medicine, Yangtze University, Jingzhou, China; 2 Department of Laboratory Medicine, First Affiliated Hospital of Yangtze University, Jingzhou, Hubei, China; 3 Clinical Molecular Immunology Center, School of Medicine, Yangtze University, Jingzhou, Hubei, China; 4 Department of Rheumatology and Immunology, Tongji Hospital, Huazhong University of Science and Technology, Wuhan, Hubei, China

**Keywords:** Type 1 diabetes, B cells, T cells, regulatory B cells, marginal zone B cells, IL-10

## Abstract

Type 1 diabetes (T1D) is a metabolic disorder caused by a complete lack of insulin, primarily manifested by hyperglycemia. The mechanisms underlying the onset of T1D are complex, involving genetics, environment, and various unknown factors, leading to the infiltration of various immune components into the islets. Besides T cells, B cells are now considered important contributors to the pathogenesis of T1D, according to recent studies. In non-obese diabetic (NOD) mice, the absence of B cells prevents the development of T1D, and B-cell depletion can even restore the function of pancreatic β cells, emphasizing their involvement in the development of T1D. Naturally, besides pathogenic B cells, regulatory B cells (Bregs) might have a protective function in T1D. This article examines the mechanisms behind B-cell tolerance and the defects in B-cell tolerance checkpoints in T1D. We explored possible functions of B cells in T1D, including the role of islet autoantibodies in T1D, T–B cell interactions, and the role of Bregs in the pathogenesis of T1D. We also summarized the advances of B cell-targeted therapy, exploring new methods for intervention and treatment of T1D.

## 1 Introduction

Type 1 diabetes (T1D) poses a significant threat to human health. When patients develop clinical symptoms, the remaining pancreatic β cells in the body are often less than 10%, leading to an absolute lack of insulin. Patients have to rely on lifelong exogenous insulin-compensating therapy to alleviate symptoms (1). The incidence of T1D is increasing among children under 15 years old, especially in the age group of 0–5 years. Multiple research centers in Europe have indicated that the annual growth rate of T1D in children under 5 years old is 5.4% (2), which causes a significant increase in the burden on healthcare systems worldwide. A phase II randomized controlled trial discovered that patients with T1D receiving rituximab, an anti-CD20 monoclonal antibody (mAb), had significantly higher serum C-peptide levels compared to the placebo group. Additionally, hemoglobin A1c(HbA1c) levels were significantly reduced, and the deterioration of pancreatic β-cell function in individuals with T1D can be markedly postponed (3). Nonetheless, follow-up research indicates that the initial therapeutic effects tend to wane once B-cell counts revert to normal levels after initial treatment (4).

Islet-reactive B cells play a crucial role in T1D pathogenesis by presenting antigens to T cells and producing cytokines and autoantibodies (5–8). In addition, the results from non-obese diabetic (NOD) mice also suggest that a fall in the number of B cells can prevent autoimmune diabetes (9–13). Furthermore, the survival rate of islet allografts was improved in mice with a shortage of B cells (14). In conclusion, there is considerable evidence suggesting that B cells have a major role in T1D. Therefore, further research into the specific mechanisms of B cells in T1D is crucial for developing new therapeutic approaches.

## 2 The existence of B cells in the process of T1D

### 2.1 Mechanisms of B-cell tolerance

B-cell polyreactivity is defined by the ability of a single B-cell receptor (BCR) to structurally bind to unrelated antigens. Research on human pre-B cells indicates that the majority of antibodies expressed by early immature B cells exhibit self-reactivity. A total of 55%–75% of newly generated B cells in the bone marrow are capable of binding to self-antigens such as DNA or insulin through their BCRs, indicating a high level of destructiveness in these cells (8). Under normal circumstances, the body regulates autoreactive B cells through various mechanisms to decrease their prevalence in the B-cell pool and their attraction to their own tissues. B-cell tolerance mechanisms are categorized into peripheral and central tolerance, each exerting distinct functions at different phases of B-cell ontogeny (15).

**Figure 1:**
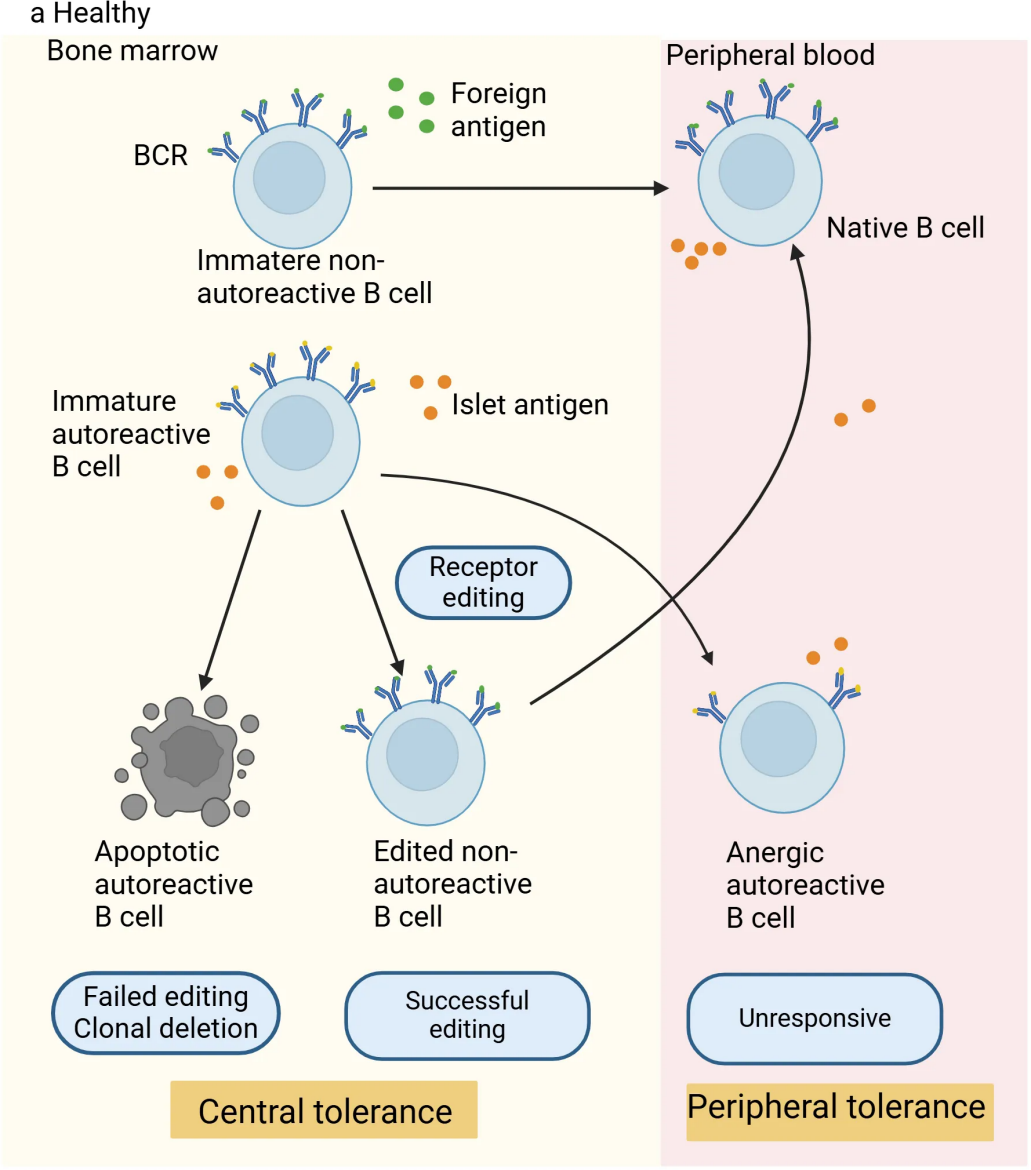
Mechanisms of B-cell tolerance in healthy individuals. In healthy individuals, non-autoreactive B cells react to foreign antigens through their B-cell receptors (BCRs), successfully migrate to the periphery, and become mature naive B cells. Autoreactive B cells that can bind to self-antigens undergo receptor editing in the bone marrow (a process considered as a central tolerance mechanism). Cells that are successfully edited migrate to the periphery, while those that fail receptor editing are destroyed. Autoreactive B cells with moderate affinity for self-antigens can enter the periphery and acquire tolerance through peripheral tolerance mechanisms, thus preventing activation, proliferation, and losing the ability to produce antibodies against self-antigens. This figure was adapted from Nat. Rev. Nephrol. (DOI: 10.1038/nrneph.2017.138) with permission from Springer Nature (License #: 6038731357027).

Receptor editing takes place within the bone marrow, and it is a process that nearly all autoreactive B cells can experience. Through secondary immunoglobulin (Ig) gene rearrangement, B cells generate specific BCRs that are harmless to the organism. The gene responsible for the Ig light chain of the BCR undergoes rearrangement by silencing one allele while expressing the other, resulting in a uniquely modified BCR (16, 17). This process does not depend on the affinity for self-antigens and the presence of non-self-reactive B cells (16). Research indicates that approximately 25% of the light chains on maturing B cells’ surfaces come from receptor editing, according to data from four separate Igk knock-in mouse strains (18), this suggests that receptor editing is crucial in shaping the standard antibody repertoire. This process eliminates the self-reactivity of many cells, and when this mechanism fails to eliminate self-reactivity, sustained signals induced by self-antigens will lead to apoptosis and death of self-reactive cells. In the central tolerance of B and T cells, apoptosis triggered by self-antigen recognition is significant, given that cells in the initial development stages are highly prone to this type of cell death (15). A group of newly generated non-self-reactive B cells move from the bone marrow to the peripheral lymphoid organs, maturing into natural B cells that carry out protective immune functions. As they cannot bind to self-antigens, they do not pose any harm to the organism. In addition to receptor-edited B cells and those unable to bind self-antigens, there is another category of B cells with moderate self-antigen affinity that can move from the bone marrow to peripheral lymphoid organs and become inactive through a peripheral tolerance mechanism called anergy (19). **Figure 1** shows the mechanisms of B-cell tolerance.

### 2.2 B-cell tolerance checkpoint defects in T1D

Research indicates that patients with T1D exhibit increased autoreactive B-cell levels compared to those without the disease (20). Self-reactive B cells are usually silenced through three mechanisms throughout their developmental process: receptor editing or apoptosis of immature B cells during central tolerance in the bone marrow, and anergy during peripheral tolerance (8). Research has shown that in patients with T1D, there is a higher occurrence of autoreactive B cells during the new migration and/or transitional and mature naive B cell phases, indicating that the mechanisms for central and peripheral B-cell tolerance are compromised in patients with T1D (20). Furthermore, these cells exhibit polyreactivity, being able to interact with single-stranded DNA, double-stranded DNA, insulin, and lipopolysaccharides (20). Since recombination sequences accumulate in receptor-edited cells and can be used for identification, a research evaluated the rate of recombination sequence rearrangements in B cells positive for λ-Ig light chains among individuals with T1D and the control group. Rearrangement of recombination sequences in B cells from patients with T1D was found to be less frequent than in healthy individuals, indicating a possible flaw in the receptor editing mechanism in patients with T1D, which could allow self-reactive B cells to migrate to the periphery (21).

Under normal circumstances, autoreactive lymphocytes are strictly regulated by tolerance mechanisms, which aids in preventing the development of autoimmune diseases. However, in patients with T1D, the interaction between genetic risk alleles and environmental risk factors can enable autophagy-active lymphocytes to evade these tolerance checkpoints, resulting in their activation and the progression of autoimmunity (22). Autoreactive B cells can produce autoantibodies targeting self-antigens, triggering the onset of autoimmune responses. Even though antibodies themselves cannot destroy β cells, autoreactive B cells pose a threat to the organism. Besides producing antibodies, they can function as efficient antigen-presenting cells (APCs) to trigger T1D. For human T1D, B cells that respond to insulin serve as APCs, promoting the activation of self-reactive CD4^+^ and CD8^+^ T cells (23, 24).

**Figure 2:**
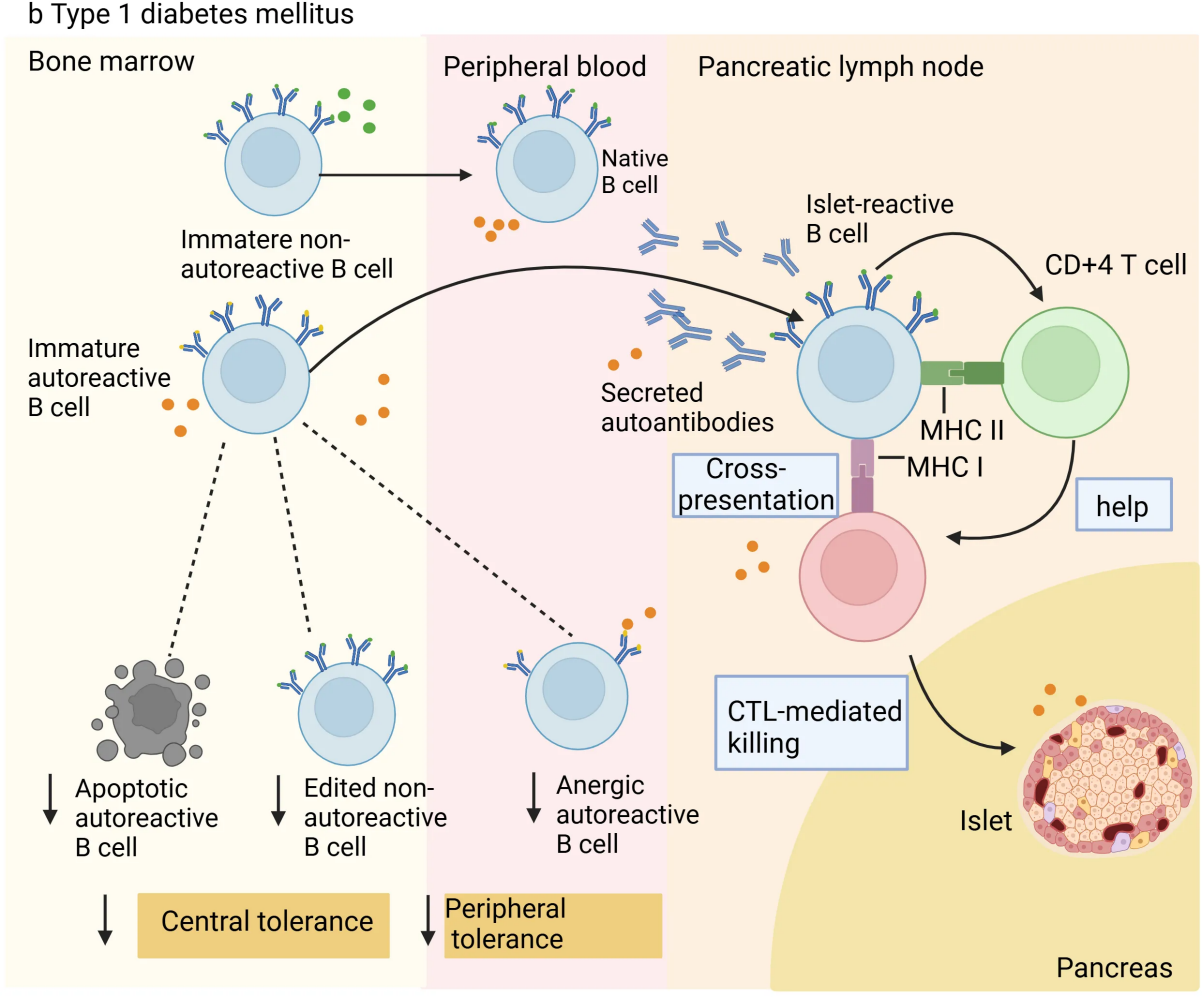
Illustration of B cell tolerance mechanism defects in patients with Type 1 diabetes. In type 1 diabetes, owing to defects in central and peripheral tolerance mechanisms, autoreactive B cells accumulate in the periphery. These cells enter the pancreas or pancreatic lymph nodes to destroy islet b cells. They can present antigens to islet-reactive CD4+ and CD8+ T cells, causing the destruction of islet b cells through cytotoxic T lymphocyte (CTL)-mediated killing, resulting in insufficient insulin release and triggering T1D. This figure was adapted from Nat. Rev. Nephrol. (DOI: 10.1038/nrneph.2017.138) with permission from Springer Nature (License #: 6038731357027).

Additionally, studies with NOD mice provide growing evidence that the primary factor in T1D is the loss of B-cell tolerance (25–27). Utilizing single-cell RNA sequencing technology, a study explored the affinity of BCRs for insulin and the interaction with B-cell phenotypic changes during disease progression. The findings suggested that the relaxation of tolerance mechanisms led to the buildup and partial activation of B cells with lineage-encoded high-affinity BCRs in NOD mice, thereby encouraging the development of autoimmunity (28). Throughout the disease progression in VH125.hCD20/NOD mice, anti-insulin B cells are attracted to the pancreas, and after receiving anti-CD20 therapy, they reappear in the islets before non-specific B cells do (29). These findings further highlight the significant role of autoreactive B cells in the development and advancement of T1D. Comprehending the mechanisms behind diseases and their progression is vital and might reveal key insights for developing innovative therapies. **Figure 2** shows the manifestation of the disruption of autoreactive B-cell tolerance mechanisms and dysfunction in T1D.

### 2.3 Possible causes of defective B-cell tolerance mechanisms in T1D

Importantly, anergy is reversible. Therefore, when anergy is disrupted, autoimmunity may follow, possibly through receiving homologous T-cell help (signal 2) and TLR stimulation (signal 3), and/or changes in inhibitory receptor signals (30, 31). Earlier research shows that the B-cell anergy is attributed to susceptible genes for insulin-dependent diabetes mellitus located on chromosome 1 (Idd5) and chromosome 4 (Idd 9/11) (32). Recent research by the Smith team has found that high-risk HLA alleles and non-HLA risk allele sets associated with the development of B and T cells and their immune functions [i.e., PTPN2 (rs1893217), INS (rs689), and IKZF3 (rs2872507)] are linked to B-cell incompetence (33). The expression of Src homology-2 (SH2)-containing inositol 5-phosphatase SHIP-1 and inositol phosphatase PTEN (phosphatase and tensin homolog) in the phosphatidylinositol 3-kinase (PI3K) pathway mediated by Bcr and cxcr4 plays an important part in keeping B-cell anergy (34, 35). Fc receptor-like 5 (Fclr5), highly expressed in autoreactive B cells, has been found to break the anergy of B cells. The upregulation of Fclr5 in B cells exacerbates SLE and enhances the expression of TLR signals, suggesting that Fclr5 is a potential regulatory factor for B cell-mediated autoimmune diseases (36). The caspase recruitment domain family member 19 (CARD9) can also negatively regulate BCR/TAK12-induced NF-κB activation, and its deficiency can enhance B-cell tolerance (37).

Studies in NOD mice found that by targeting the PI3K pathway, recent studies have revealed that p110δ inhibitors can compensate for the dysregulation of the PI3K pathway in autoimmune mouse models. It has also been discovered that low doses of the p110δ inhibitor Idelalisib can prevent the body from mounting an immune response to exogenous antigens, thus preventing the development of autoimmune reactions driven by acute induction of B cell-targeted deficiency in PTEN and SHIP1, which impairs PI3K pathway regulation. Nevertheless, they cannot prevent autoimmune reactions induced by B cells losing regulation of the tyrosine phosphatase SHP-1. Furthermore, it was observed that using a low-dose p110δ inhibitor in NOD mice can focus on the lowered PTEN expression in B cells, stopping the advancement of the disease in the T1D model, which contributes to the development of precise therapeutic approaches (38). Additionally, monoclonal IgMs from healthy donors reduced the self-reactivity of B lymphocytes, enhanced the generation of regulatory T cells (Tregs), and reversed T1D in NOD mice, indicating that naturally produced Igs may also emerge as a viable treatment option for T1D (39).

Central to disease development is the breakdown of B-cell tolerance, making it crucial to understand how autoreactive B cells become non-anergic. This knowledge can aid in developing innovative therapies for T1D, like altering the immune regulation by B cells. This might aid in halting the progression of T1D or mitigating its severity.

## 3 Potential effector mechanisms of B cells in T1D

### 3.1 The role of islet autoantibodies in the pathogenesis of T1D

Existing evidence indicates that the presence of islet autoantibodies is one of the best predictors of T1D development (40). The peak of autoantibody production occurs during childhood, and they can be identified as early as 6 months old but can continue to develop throughout life (41, 42). In T1D, five specific autoantibodies targeting antigens have been identified: insulin autoantibodies (IAA), glutamic acid decarboxylase antibodies (GADA), islet antigen-2 antibodies (IA-2A), zinc transporter 8 antibodies (ZnT8A), and tetraspanin-7 antibodies (Tspan7A) (43). Among these, the most common is the specificity against insulin (44), and then there may gradually appear autoantibodies against other major pancreatic islet cell antigens, including GAD, IA-2A, and ZnT8A (45).

Many early studies on NOD mice have shown that autoantibodies may not be especially critical in the progression of autoimmune diabetes. Neither transferring human serum into SCID mice nor eliminating maternal autoantibodies postnatally through breastfeeding affected the incidence of T1D, indicating that without B cells, autoantibodies alone cannot affect the occurrence of T1D (46, 47). Moreover, experiments on NOD mice found that NOD mice with B-cell deficiencies that expressed mutated heavy-chain Igs on their cell surface but could not secrete mIgs had a greater occurrence of insulitis and diabetes compared to B cell-deficient NOD mice that are transgene-negative. This suggests that B cells’ capacity to generate antibodies does not necessarily affect the development of diabetes, and CD138^+^ plasma cells were seldom detected within the target organs (48). However, many observations suggest that autoantibodies may influence the disease’s pathogenesis. In NOD mice, autoantibodies can exert pathogenic effects through Fc receptor-mediated antigen-antibody uptake and subsequent activation by dendritic cells (DCs) and macrophages, resulting in the activation of autoreactive T cells (49). B cells secrete antibodies against pancreatic autoantigens, which can boost the growth of pancreatic-reactive CD4^+^ T cells through FcγR-mediated processes, contributing to T1D (50).

Research in patients with T1D has also found that islet autoantibodies secreted by B lymphocytes are typically considered indicators of the disease rather than a pathogenic factor (40, 51). Autoantibody titers are intimately associated with the complications of T1D (52, 53), and patients with high titers of antibodies are at a greater risk of suffering from other autoimmune diseases or being positive for autoimmune-related antibodies. In addition, pancreatic autoantibodies (including GADA, IA-2A, and ZnT8A) can predict the risk of T1D, increasing the sensitivity of diagnosing patients with T1D (54, 55). According to a study from May, the initial appearance of certain autoantibodies can affect the early immune response in children who develop clinical diseases, with those having two or more autoantibodies showing elevated levels of CD161 in natural killer cells (56). Another study used a phenotype comprising 25 regions formed by the five areas under the glucose curve (AUCGLU) and the five areas under the C-peptide curve (AUCPEP) to gain deeper insights into the heterogeneity of T1D. It was found that the 5-year risk of T1D is highly associated with the antibodies mIAA and IA-2A (57, 58).

In essence, the preclinical period of T1D is defined by the detection of serum islet autoantibodies, indicating that the immune system has commenced its assault on the islet β cells. Therefore, islet autoantibodies in the serum can help predict the onset of T1D and allow for early intervention before clinical symptoms appear. Gaining a clearer insight into the connection between autoantibody presence, insulitis, and cell damage will help in creating improved treatments to prevent or cure the disease.

### 3.2 The role of T–B cell interactions in T1D

T1D is marked by the destruction of pancreatic β cells, mainly driven by CD4^+^ and CD8^+^ T cells, resulting in inadequate insulin production and a lifelong need for external insulin therapy to alleviate symptoms (59). Cytokine secretion by Th1 and Th17 cells is thought to lead to β-cell apoptosis, while Th2 and Treg cells provide protective functions; studies also reveal T-cell imbalance in the peripheral blood of patients with T1D (60–62). CD40 is identified as the latest indicator for a specific pathogenic T-cell subset in T1D, with established Th1 or Th2 phenotypes of diabetic T cells being CD40-positive (63). Furthermore, the cascade of reactions triggered by physiologic pancreatic islet β cells, including B-1a cells, neutrophils, and IFN-α-secreting plasmacytoid dendritic cells (pDCs), plays a critical role in initiating T1D-related T-cell responses and driving their progression (64).

#### 3.2.1 B cells and CD4^+^ T cells: role in antigen presentation

It is widely recognized that B cells efficiently present antigens to CD4^+^ T cells during the development of T1D (5, 65, 66). B cells, as crucial APCs, play a role in the development of T1D by expressing high levels of MHC class I and II molecules, co-stimulatory molecules (66, 67), and autoantibody secretions (50), and secreting various cytokines (68, 69). CD4^+^ T cells support B cells and promote the activation of humoral immune responses. After maturing, B cells are produced in the bone marrow and move to the pancreas during the initial phases of inflammation (70). This subset of immune cells infiltrating the pancreas remains dormant due to the influence of the surrounding inflammatory environment, acting as APCs here to induce the proliferation of T cells infiltrating the pancreas, leading to subsequent autoimmune reactions (71). In conditions where antigens are limited, T cells from NOD mice show greater proliferation when stimulated by B cells *in vitro* compared to other APCs, suggesting that B cells are the favored APCs for autoreactive T cells (6, 72). Early researchers established NOD mice deficient in B-cell compartment I-Ag7 and found that B cell-mediated Ag presentation of I-Ag7 facilitates overcoming the immunological tolerance checkpoint of pancreatic β cells by T cells after initial targeting. To sum up, the complete autoimmune function of anti-pancreatic β-cell T cells in NOD mice depends on the accurate control by B cell-mediated MHC II antigen presentation (5). These conclusions also demonstrate that in T1D, compared to other APCs, B cells are considered the primary APCs for T cells.

Recent research indicates that B cells in the plasmablast stage, which secrete antibodies, can present antigens to CD4^+^ T cells, leading to their activation and triggering pro-inflammatory responses, thereby worsening T cell-mediated β-cell destruction. Intravenous injection of plasmablasts along with NOD mice T cells into NOD/SCID mice accelerated the onset of disease in NOD/SCID mice (73). TLRs can mediate endogenous immune responses, playing a crucial role in T1D. According to recent research, NOD mice without Tlr7 (Tlr7^−/−^) show a significantly postponed onset and decreased rate of T1D compared to those with adequate Tlr7 (Tlr7^+/+^). The mechanism shows that B cells from (Tlr7^−/−^) NOD mice suppress the CD4^+^ T-cell response to diabetes, protecting immunodeficient NOD mice from diabetes induced by diabetogenic T cells. Moreover, lacking Tlr7 hampers B cells’ ability to present antigens and reduces the activation of cytotoxic CD8^+^ T cells by decreasing the expression of both classical and non-classical MHC class I molecules on B cells (74). In NOD mice, the absence of B-cell TLR9 can postpone the onset of T1D by altering the frequency and function of various B-cell subsets (75).

2H6 is an insulin-reactive CD4^+^ T cell that protects NOD mice from TGF-β-mediated T1D development. A study involved crossing 2H6 TCR transgenic NOD mice with VH125 BCR transgenic NOD mice to produce 2H6VH125 NOD mice. The promotion of B-cell tolerance by 2H6 T cells was observed through a reduction in insulin-reactive B cells, decreased expression of B cell MHC and costimulatory molecules, and an increase in non-insulin-specific IgG expression. In short, CD4^+^ T cells convert pathogenic B cells into tolerant cells (66). A recent experiment subjected lipopolysaccharide-treated splenic B cells to electroporation, followed by treatment with mRNA encoding chimeric MHC-I or MHC-II molecules. The results showed that e-B cells with chimeric MHC-I inhibit the cytotoxicity of CD8^+^ T cells, while e-B cells with chimeric MHC-II induce CD4^+^ T cells to express regulatory markers. Additionally, these e-B cells protect NOD/SCID mice against autoimmune diabetes. This feature of engineered B cells opens up new opportunities for providing antigen-specific regulatory or pathogenic cell-clearing functions (76). In summary, these research findings provide new directions for drug development or therapeutic approaches from the perspective of T–B cell interactions.

#### 3.2.2 B cells and CD8^+^ T cells:B cells infiltrating the islets synergize with CD8^+^ T cells

In the early stages of T1D, “islet inflammation” occurs, attracting many immune cells to the islets (77). A layer of extracellular matrix (ECM), made up of the basement membrane and interstitial matrix, encloses the islets. This ECM layer acts as a divider between endocrine and exocrine tissues and is an essential barrier preventing immune cells from invading the islets and destroying β cells. Studies on T1D development in NOD mice models and human patients indicate that the BM and IM components surrounding the islet infiltrated by white blood cells are overall absent in these subjects (78). This also demonstrates that immune cells have infiltrated the islets. Up to 80% of the lymphocytes infiltrating the islets are T cells (with the majority being CD8^+^ T cells) (79). Research has demonstrated that B cells are involved in the infiltration of immune cells in the islets of both mice and humans. They are detectable in NOD mice starting at 4–7 weeks of age, with approximately 10% of islets infiltrated by then. In mice aged 8–11 weeks, islet infiltration continues to rise, reaching 50% to 60%. Typically, these cells develop tertiary lymphoid follicles that have distinct T- and B-cell sections (70). Furthermore, the B cells here do not function by secreting autoantibodies, so we need to discuss other possible roles they may play.

While both CD4^+^ and CD8^+^ T cells infiltrate the islets, β-cell destruction is primarily attributed to CD8^+^ T cells (80). HLA-A2 tetramers carrying the main T-cell epitope peptides from pancreatic self-antigens were used to detect single and multiple CD8^+^ T cells reactive to self-antigens within the islets in frozen sections of the T1D pancreas (81). Tfh cells are capable of secreting different cytokines to enhance B-cell proliferation and activation, and B cells, in turn, seem to significantly influence T cells. Research conducted in B cell-deficient NOD mice demonstrates that B cells not only act as APCs to assist CD4^+^ T cells in responding to islet antigens during the progression of T1D but also contribute to promoting the survival of islet-reactive cytotoxic CD8^+^ T cells (82).

Research on NOD mice revealed that besides attacking islet β cells, the absence of B cells also hindered the differentiation of CD8^+^ T cells into cytotoxic T lymphocytes (CTLs) (83). In the development of autoimmune disease like T1D, autoreactive CD8^+^ T cells need to engage with antigen peptides displayed on APC surfaces, mainly in a manner restricted by MHC class I, and this interaction regulates the transition from clinically asymptomatic insulitis to asymptomatic diabetes (66). The elimination of B cells post-insulitis offers protection against T1D, though it results in less activation of CD8^+^ T cells (9). When B cells are depleted early, T cells infiltrating the islets show a lack of activation, especially with reduced CD44 expression and effector function. It also affects the ability of CD4^+^ T cells and CD8^+^ T cells to secrete IFN-γ, and these CD8^+^ T cells continue to change within the islets for a long period after the depletion and repopulation of B cells (84). These results prompt us to consider the existence of a close connection between B cells and CD8^+^ T cells.

Research investigated the distribution and morphological alterations of CD20^+^ cells within the pancreas and discovered that B lymphocytes undergo notable morphological changes when migrating through the pancreas, and these changes are associated with their positioning with CD8^+^ T cells and target islets. As the relationship between B cells and islets becomes tighter, particularly upon encountering CD8^+^ T cells, the clustering of CD20 antigen on the cell surface intensifies (85). This discovery indicates that T cells are not activated in the localized islet infiltration following early B-cell depletion (86), suggesting a collaborative role of CD8^+^ T lymphocytes with B cells in human T1D. This highlights the intricate nature of T1D, necessitating a thorough examination of the roles of various immune cells and molecules to enhance understanding of its pathogenesis and devise treatment approaches.

### 3.3 The impact of regulatory B cells on the development of T1D

The concept that B cells could perform regulatory functions was initially suggested in 1974 (87). The term “Bregs” (regulatory B cells) was introduced in 1997 (88). Bregs possess anti-inflammatory effects and inhibit autoimmunity through the secretion of cytokines and cell contact-mediated mechanisms. The name Bregs comes from their production of anti-inflammatory cytokines with regulatory functions. Various types of Bregs have been identified so far, with overlapping immune markers or cytokine production. Other subgroups are also gradually being revealed and summarized. **Table 1** summarizes the different phenotypes of Breg subpopulations and their origins identified to date. Mouse experiments have also demonstrated an upregulation of different phenotypes of Bregs in autoimmune diseases, all of which secrete IL-10 and play immunomodulatory roles (89). IL-10 is considered a hallmark of Bregs (90), but its expression is relatively low at the resting cellular level, and it is only significantly expressed after B-cell stimulation; hence, there is currently a lack of a very precise marker for Bregs. Moreover, the surface markers of B cells change after *in vitro* stimulation, which further complicates the identification of Bregs. Thus, the discovery of additional markers for Bregs would be a significant advancement in this research field.

**Table 1:** Different Breg cell subsets.

Types of Breg cell	Phenotype	Species	Citation
Transitional B cells	CD19^+^CD24^hi^CD38^hi^	Human	(127)
B10 cells	CD24^hi^CD27^+^	Human	(90)
	CD19^+^ CD5^+^ CD1d^hi^	Mice	(128)
Br1 cells	CD19^+^ CD25^hi^CD71^hi^	Human	(129)
Plasmablasts	CD19^+^ CD27^int^CD38^+^	Human	(130)
	CD138^+^ CD44^hi^	Mice	
	IgA^+^ CD138^+^ PD-L1^−^ IL−10^+^	Human	(131)
	CD19^+^ CD138^+^	Mice	(132)
	IgM^+^ CD138^hi^TACI^+^ CXCR4^+^ CD1d^int^Tim1^int^	Mice	(104)
	LAG3^+^ CD138^hi^	Mice	(93)
GrB^+^ B cells	CD19^+^ CD38^+^ CD1d^+^ IgM^+^ CD147^+^	Human	(133)
CD9^+^	CD19^+^ CD9^+^	Human, mice	(134)
CD19^+^	CD19^+^ CD5^+^CD1d^hi^	Human	(135)
MZ cells	CD19^+^ CD21^hi^CD23^−^	Mice	(136)
B1a cells	CD1d^hi^CD5^+^	Mice	(100)
Tim−1 ^+^ B cells	CD19^+^Tim-1^+^	Mice	(137)
Killer B cells	CD19^+^ CD5^+^ FasL^+^	Mice	(138)

B2 cells, B1 cells, and marginal zone B cells can all serve as sources of Bregs. Different types of B cells play regulatory roles under varying immune environments and stimuli (91, 92). Recent studies have found that phosphatidylcholine-reactive (PtC) B-1 cells produce regulatory phosphatidylcholines (PCs), which serve as a major source of the immune-suppressive cytokine IL-10 *in vivo* (93). IL-1β and IL-6, as well as TLR signals including various inflammatory environments, can foster the generation of Bregs (94, 95). Recent studies indicate that plasma cells are the primary Bregs subset responsible for IL-10 production and that Ca^2+^ signaling also promotes IL-10 expression in B cells (96). Interferon regulatory factor-4 (IRF4) has been discovered to positively regulate the generation of IL-10-producing plasma cells in draining lymph nodes (dLNs) (97), while Blimp-1 has also been identified as a regulator of Breg generation, differentiation, and IL-10 production (98).

**Figure 3:**
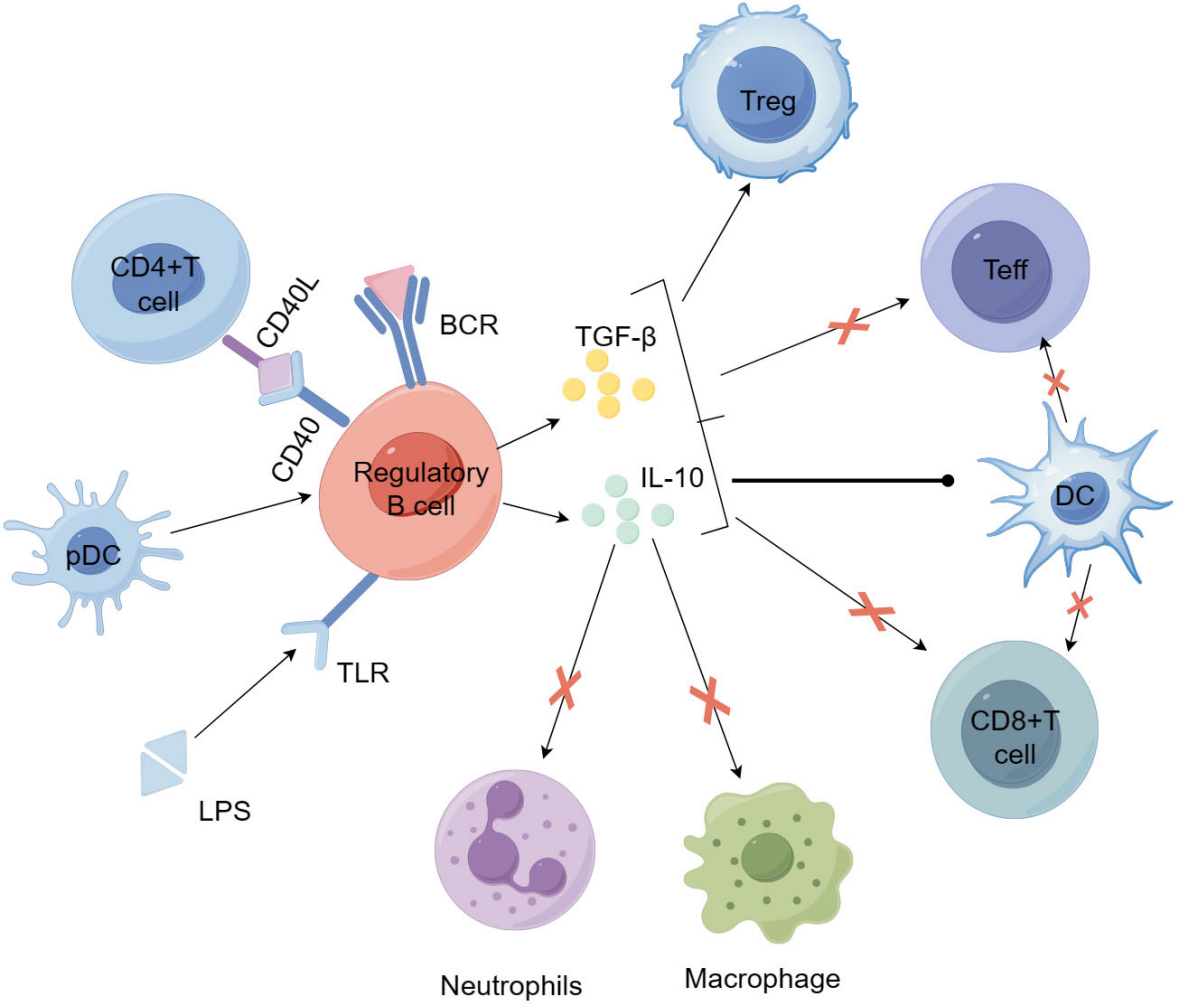
Bregs participate in the mechanisms of self-reactive T cell-mediated β-cell destruction and their effects on other immune cells. Bregs can be induced by stimulation with LPS, anti-IgM F(ab')2 antibodies, CD40L, and plasmacytoid dendritic cells (pDCs). Activated Bregs produce IL-10 and TGF-β, suppress the inflammatory potential of effector T cells, alter the activity of antigen-presenting dendritic cells, and promote the development and expansion of regulatory T cells. Bregs can further inhibit the activity of macrophages and neutrophils by secreting IL-10 and other anti-inflammatory factors, thereby reducing their pro-inflammatory responses and tissue damage. BCR, B-cell receptor; LPS, lipopolysaccharide; MHC, major histocompatibility complex; TCR, T-cell receptor.

Earlier studies have indicated that IL-10 generated by B cells is crucial in controlling autoimmune reactions (91). In both *in vitro* and *in vivo* settings, human and murine IL-10 exhibit multiple roles, including suppressing Th1 and Th2 polarization, reducing antigen presentation by DCs, monocytes, and macrophages, and enhancing the generation of pro-inflammatory cytokines (99). At present, even though the mechanism of Breg action is not completely understood, studies indicate that they primarily, but not solely, control the expression of effector T cells and APCs via IL-10 (97, 100–102). IL-10 has become a central focus of many studies investigating why Bregs fail to suppress inflammation in autoimmune conditions. Besides generating IL-10, Bregs also have immune regulatory functions by producing TGF-β and IL-35 (103, 104). The function of Bregs in T1D is evident in their capacity to uphold tolerance towards pancreatic islet self-antigens (105, 106). **Figure 3** illustrates how Bregs are involved in the self-reactive T cell-driven destruction of islet β cells and their influence on other immune cells. Although the specific role of Bregs in T1D remains under investigation, it has been demonstrated that Bregs exert a positive protective effect in T1D. Recently, review articles have summarized the involvement of Bregs in autoimmune diseases, including T1D (107).

#### 3.3.1 The protective role of Bregs in T1D

##### 3.3.1.1 Through an IL-10-dependent mechanism

The previously mentioned Bregs most commonly produce the cytokine IL-10. Tedder’s research group found a similar subset of B10 cells responsive to IL-10 in human blood, CD19^+^CD5^+^CD1d^hi^ cells, constituting 1% to 3% of the spleen’s total B-cell population (90).

In NOD mice with pancreatitis and diabetes, there is a greater number of B10 cells compared to those with normal blood glucose levels. The amount of IL-10 secreted by B10 cells in NOD mice is notably reduced. It indicates that B10 cells’ number and function could contribute to T1D’s development and progression (108). Similarly, a study using the IgVH transcriptome found that in long-term hyperglycemic NOD mice, compared to hyperglycemic NOD mice, the levels of CD40^+^ B cells promoting Breg and IL-10-producing B cells increased in the islets (124). BCR-activated B cells administered intravenously can shield NOD mice from T1D effects through an IL-10-dependent mechanism (69). A new study highlighted the significance of the interaction between DC and Breg in T1D development. Following activation by the innate immune receptor TLR4, B cells that initially lost their IL-10 production capability could recover this function. Through a mechanism involving DCs and mediated by IL-10, they were able to suppress insulin-specific CD8^+^ T cells. Interaction between B cells and DCs resulted in DC deactivation, inducing a state of tolerance, which could also regulate pathogenic CD8^+^ T cells in turn (109).

The healthy control group exhibits a greater number of CD40^+^ B cells and IL-10^+^ B cells compared to patients with T1D (105). Compared to adults with LADA and patients with T2D, patients with T1D have a reduced presence of B10 cells (110). These experimental findings collectively demonstrate a reduced frequency of B10 cells in patients with T1D. These experimental results collectively indicate a decreased frequency of B10 cells in patients with T1D. Moreover, the proportions of IL-10^+^CD24^hi^CD27^+^ Bregs cells (B10) and IL-10^+^CD24^hi^CD38^hi^ (immature transitional) Bregs cells in the circulation of pediatric patients with T1D are notably reduced compared to the healthy control group (111). IL-10^+^ B cells extracted from patients with T1D show stronger immunosuppressive activity. They are capable of suppressing autoimmune reactions initiated by autoreactive T cells when the pancreatic islet antigen mimics epitope IA-2 is present (109). These findings also provide important insights for the exploration of therapeutic strategies for T1D based on B10 cells. The study of B-cell immune profiles in patients with T1D identified a subgroup of CD24^hi^CD38^hi^ B cells, known as transitional B cells, which secrete IL-10 (125). This subset was found to exhibit regulatory properties, and deficiencies in this cell subset were observed in patients with T1D (106).

As previously stated, the IL-10-dependent role of Bregs is significant in T1D, assisting in the prevention of autoimmune reactions, enhancing immune tolerance, alleviating inflammatory responses, and maintaining immune balance. These processes help to decelerate the damage to pancreatic β cells and hinder the onset and advancement of the disease. Therefore, increasing the production of IL-10 in Bregs could provide a new potential strategy for treating T1D.

##### 3.3.1.2 Through an IL-10-independent mechanism

Apart from the IL-10-dependent mechanism, Bregs can also suppress inflammation by promoting immune regulation and inhibiting the differentiation of pro-inflammatory T cells. They are capable of inhibiting T cell-driven inflammatory reactions and provide substantial protection in systemic autoimmune conditions such as lupus (100–102). Bregs have the potential to modulate the activity and function of other immune cells via direct interactions.

After introducing lipopolysaccharide-activated B cells into NOD mice before they develop diabetes, these cells begin expressing Fas ligand and secreting TGF-β, which can inhibit Th1-mediated autoimmune responses without promoting Th2 responses to β-cell autoantigens (68). Ratiu et al. demonstrated that modulating the AID/RAD51 axis can induce the expansion of CD73^+^ Bregs in mice, protecting NOD mice from T1D. This protective effect is primarily through the inhibition of diabetogenic T-cell immune responses or the limitation/elimination of autoreactive B lymphocytes, relying mainly on CD73-mediated adenosine production (112). Studies suggest that adenosine production mediated by Bregs cells may be more critical in immunosuppression than IL-10 secretion. B lymphocytes require strong stimulation to produce IL-10, whereas CD73^+^ B lymphocytes can constitutively produce adenosine in the presence of a substrate (113). Bregs mentioned earlier can also produce IL-35 to function. In mice with hyperglycemia caused by multiple low-dose streptozotocin (MLD-STZ), administering IL-35 decreased the percentage of IFN-γ^+^ cells within Bregs cells, suggesting that IL-35^+^ Bregs have a protective function in T1D (114).

The discovery of different phenotypes of Bregs and signaling molecules that stimulate B-cell differentiation into Bregs is of great significance for targeting Bregs as therapeutic targets for autoimmune diseases. The study of methods to induce Bregs cells is restricted because of their rarity and the absence of distinct transcriptional markers. For example, after CD20 depletion therapy, which has been shown to have a relieving effect on T1D, there were no IL-10^+^ Bregs in the peripherally reconstituted B cells (84). It is important to mention that the non-IL-10-dependent mechanism of Bregs cells is still under investigation and has not been fully elucidated. Further investigation and exploration can lead to a more comprehensive understanding of Bregs mechanisms in T1D, establishing a theoretical groundwork for developing novel treatment strategies.

In general, Bregs serve an essential function in T1D through various mechanisms such as inhibiting autoimmune reactions, promoting immune tolerance, and suppressing inflammatory responses. We must further understand the relative proportion and balance between effector B cells and Bregs to improve immune therapies targeting these lymphocytes, including using Bregs as treatment options, selective B cell-targeted therapies, specific elimination of pathogenic B cells, and enhancing Breg production in patients with T1D.

## 4 B cell-targeted therapy

With the in-depth research on the role of B cells in the pathogenesis of T1D, B cell-targeted therapy takes the stage. Initially confirmed in mice, short-term depletion of B cells by targeting CD20 mAb can achieve long-term prevention and, in some cases, can reverse the occurrence of hyperglycemia in NOD mice (9, 11). Following this, these findings were confirmed in patients with T1D, showing that rituximab infusion therapy, which depletes B lymphocytes, can delay the loss of β-cell function in newly diagnosed patients with T1D (3). However, Phase II studies of these drugs have demonstrated that this effect is temporary. In the initial 6 months after treatment, B cells were greatly reduced, but they returned to normal levels between 12 and 18 months posttreatment. Patients with T1D who received rituximab had lower insulin needs and maintained C-peptide levels 1 year after treatment, but this benefit was not sustained at the 2-year mark (4). It is crucial to recognize that numerous newly diagnosed patients who have not received treatment show stable C-peptide levels as the disease naturally progresses (115). Following rituximab therapy, B cells temporarily decline, whereas T cells increase (115). Studies have shown that IgM antibodies decrease 1 year after treatment and take more than a year to recover. During this period, B cells’ response to new antigens is reduced, but IgG responses remain unchanged. After B cells recover, they can react to both familiar and novel antigens, with naive B cells regaining function more quickly than memory B cells (116). Rituximab cannot repair the defects in early B-cell tolerance checkpoints, and the number of autoreactive and polyreactive B cells remains unchanged after treatment (117).

Research suggests that following rituximab therapy, there is a decline in circulating Tfh cells and serum IA2A levels, as well as a decrease in IL-21, IL-6, and Bcl-6 mRNA expression (118). Following CD20 antibody treatment in NOD mice, Th17 cells and IL-17A levels in the spleen and pancreas were reduced, and Treg cell levels increased. Anti-CD20 treatment may relieve insulitis by modulating the Th17/Treg cell and pro-inflammatory/anti-inflammatory balance, thereby benefiting β-cell function (119). Research showed that following anti-CD20 therapy, anti-insulin B cells returned to the islets sooner than non-specific B cells, and there was a notable rise in CD138^+^insulin^+^CD19^−^ B cells after B-cell depletion (29). Previous research has indicated that the presence of CD20 molecules in all B cells diminishes when they reach the islets (119). Therefore, this group of anti-insulin B cells might be the reason for the delayed loss of C-peptide in patients with T1D following anti-CD20 treatment (29). Overall, although rituximab has shown significant therapeutic effects in some autoimmune diseases, its effectiveness in treating T1D is not ideal due to the complexity of the disease and the interplay of multiple factors.

Recent research indicates that depleting B cells and activating Tregs improves treatment results for T1D. A study compared the effects of CD4^+^CD25^hi^CD127^−^ Tregs alone versus in combination with rituximab. The results indicated that at 24 months, both treatment groups had a superior C-peptide area under the curve (AUC) compared to the control group, but only the combination therapy improved this metric at 12 and 24 months. Moreover, the frequency of remission periods at 3, 6, 9, and 21 months was significantly greater in the group receiving combination treatment than in the control group. Overall, the combination therapy with rituximab outperformed monotherapy in terms of C-peptide levels and maintaining remission over 2 years, effectively delaying the progression of T1D (120). Researchers investigated the effects of combining autologous Treg with anti-CD20 treatment on immune parameters. The findings showed that the group receiving combination therapy had elevated IgG2 levels and a greater percentage of Bregs, potentially leading to improved clinical results. The effectiveness was linked to an increase in PD-1^+^ T cells and the restoration of a B-cell tolerance phenotype (121). The study demonstrated that culturing β-like cells expressing CD19 alongside CD19 CAR-T cells led to T cell-mediated β-like cell death and T-cell cytokine release. Among them, PDL1 is one of the most upregulated genes (122). The results suggest that PDL1 could emerge as a promising target for T1D treatment, and the combination therapy of rituximab and Treg might offer a new therapeutic strategy.

## 5 Discussion

This article summarizes the tolerance mechanisms of B cells and B-cell tolerance checkpoint defects in T1D, as well as the potential pathogenic mechanisms of B cells in T1D, including the secretion of autoantibodies by autoantibody-secreting cells, serving as APCs presenting self-antigens to CD4^+^ T cells, participating in coexistence with CD8^+^ T cells around the islets, participating in early regulation of the pancreatic microenvironment in disease, and differentiating into Bregs to exert a protective effect against T1D. A more thorough investigation into the pathogenesis of B cells in T1D is advantageous for the discovery of new therapeutic approaches. Apart from the peripheral blood and pancreatic infiltrating B cells discussed, B cells are also found in the thymus of mice and humans, linked to the central tolerance of T cells. Recent research has identified that B cells in the thymus (123) also engage in the development of T1D. Quantifying thymic plasma cells, it was observed that Igs bound *in situ* to a certain proportion of medullary thymic epithelial cells (mTECs) with undefined antigens, and this binding was associated with the apoptosis of mTECs, including cells expressing insulin (124). This implies that the thymus could also be a significant target of autoimmune response in T1D, influencing the progression of the disease. To understand the pathogenesis of T1D and identify the best immune therapy strategies, it is essential to conduct a detailed analysis of the different B-cell subsets involved in the disease’s progression. Apart from the initially highlighted T cells and subsequently B cells in T1D, recent research has slowly revealed the contribution of a series of reactions of the primary target, pancreatic islet β cells, to T1D, such as β-cell stress and aging (125, 126). Further research into the mechanisms of immune tolerance in T1D indicates that immune therapy may reverse and slow the progression of T1D. Hence, additional studies on the mechanisms behind immune imbalance and its triggering factors that drive autoimmunity may uncover new targets for immune therapy. We hope that the living standard of patients with T1D can be improved in the future, allowing them to no longer rely on insulin treatment.
