# Correction: The role of B cells in the pathogenesis of type 1 diabetes

**DOI:** 10.3389/fimmu.2026.1875071

**Published:** 2026-05-29

**Authors:** Ya-nan Wang, Ruihua Li, Yaxuan Huang, Hui Chen, Hao Nie, Lian Liu, Xiaoting Zou, Jixin Zhong, Bing Zheng, Quan Gong

**Affiliations:** 1 Department of Immunology, School of Medicine, Yangtze University, Jingzhou, China; 2 Department of Laboratory Medicine, First Affiliated Hospital of Yangtze University, Jingzhou, Hubei, China; 3 Clinical Molecular Immunology Center, School of Medicine, Yangtze University, Jingzhou, Hubei, China; 4 Department of Rheumatology and Immunology, Tongji Hospital, Huazhong University of Science and Technology, Wuhan, Hubei, China

**Keywords:** Type 1 diabetes, B cells, T cells, regulatory B cells, marginal zone B cells, IL-10

There was a mistake in the captions of **Figures 1**, **2** as published. The figures were similar to prior work in Nature Reviews Nephrology (DOI: 10.1038/nrneph.2017.138) without an appropriate citation or copyright permission statement. The corrected captions of **Figures 1**, **2** appear below:

**Figure 1:**
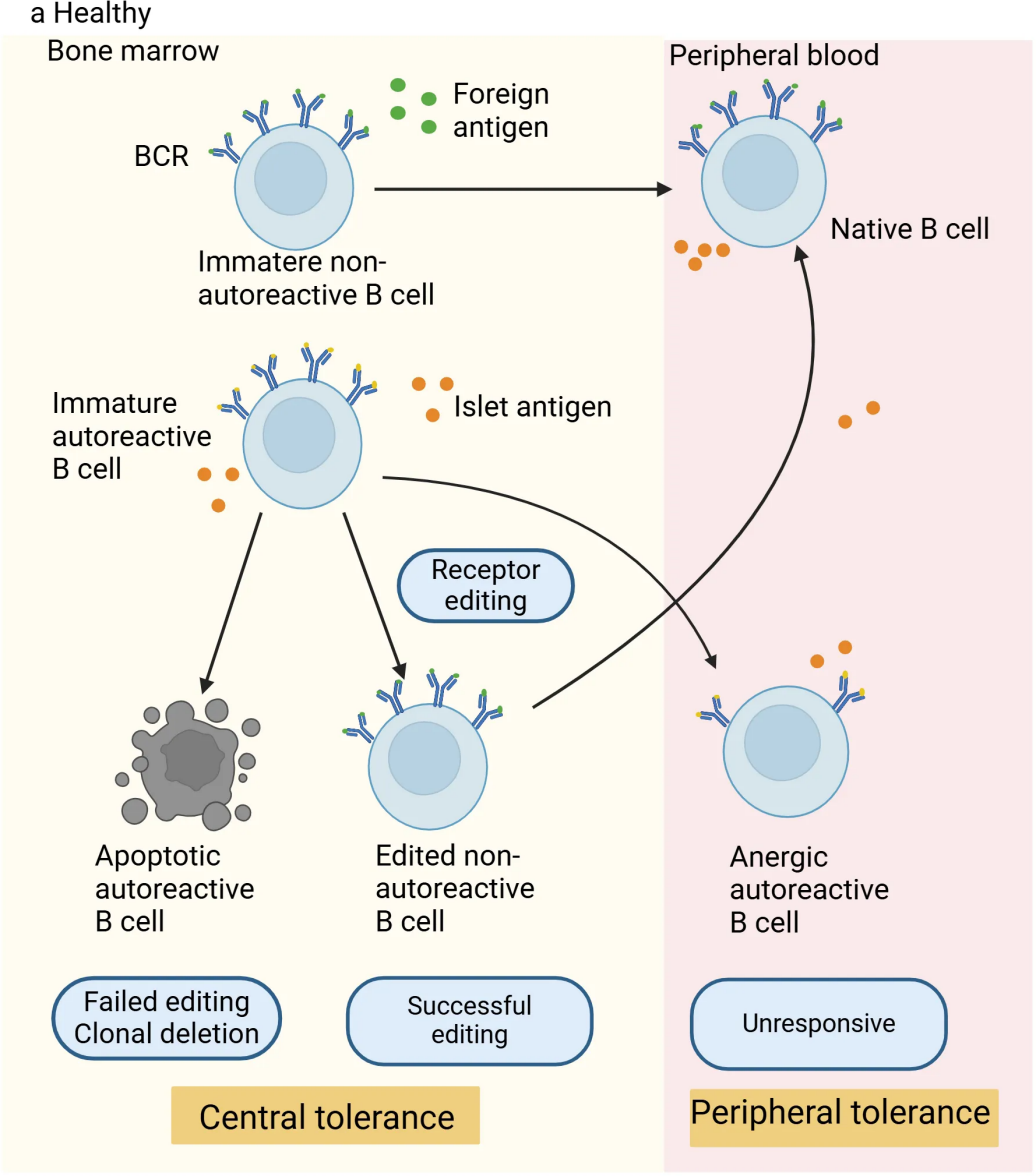
Mechanisms of B-cell tolerance in healthy individuals. In healthy individuals, non-autoreactive B cells react to foreign antigens through their B-cell receptors (BCRs), successfully migrate to the periphery, and become mature naive B cells. Autoreactive B cells that can bind to self-antigens undergo receptor editing in the bone marrow (a process considered as a central tolerance mechanism). Cells that are successfully edited migrate to the periphery, while those that fail receptor editing are destroyed. Autoreactive B cells with moderate affinity for self-antigens can enter the periphery and acquire tolerance through peripheral tolerance mechanisms, thus preventing activation, proliferation, and losing the ability to produce antibodies against self-antigens. This figure was adapted from Nat. Rev. Nephrol. (DOI: 10.1038/nrneph.2017.138) with permission from Springer Nature (License #: 6038731357027).

**Figure 2:**
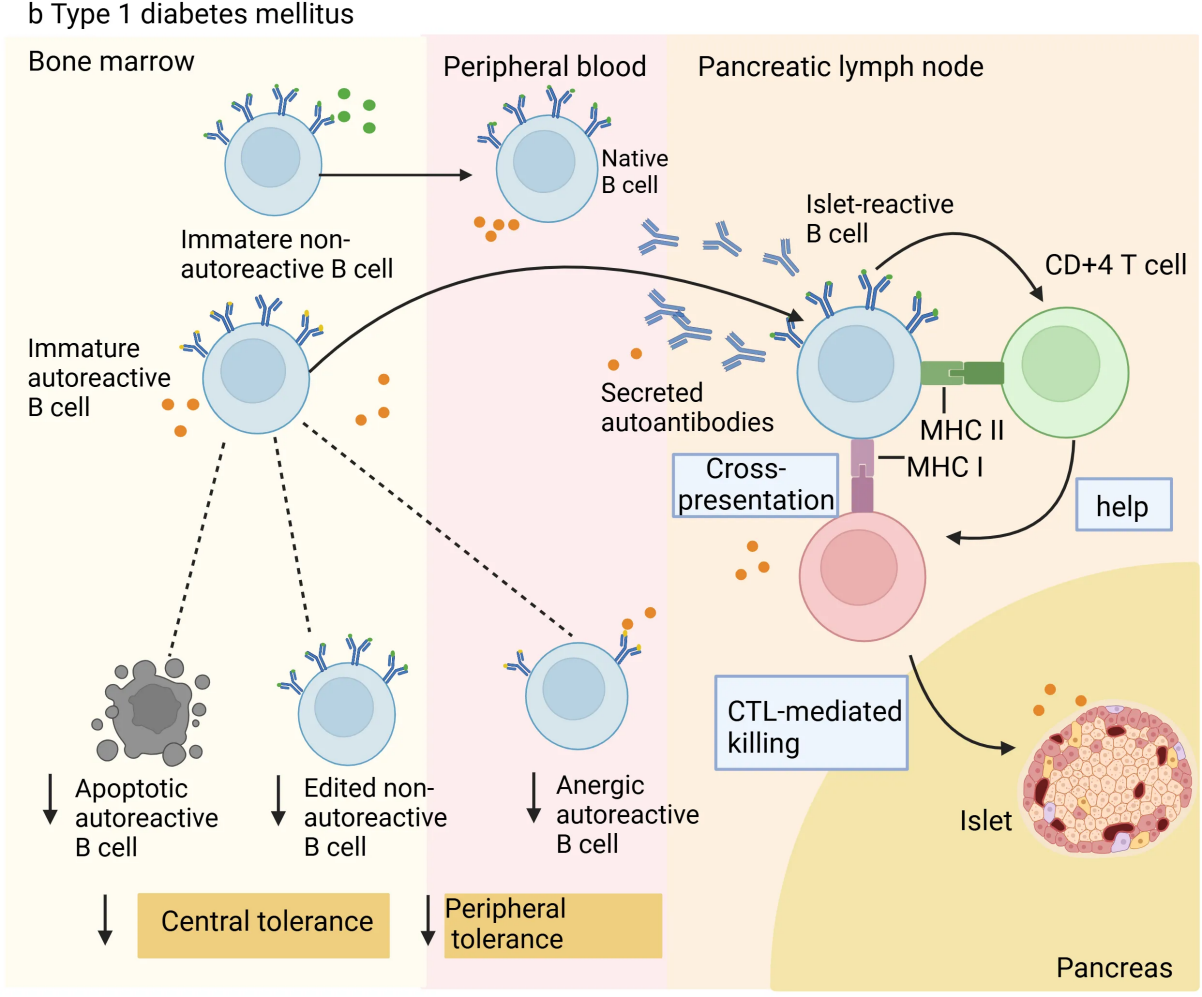
Illustration of B cell tolerance mechanism defects in patients with Type 1 diabetes. In type 1 diabetes, owing to defects in central and peripheral tolerance mechanisms, autoreactive B cells accumulate in the periphery. These cells enter the pancreas or pancreatic lymph nodes to destroy islet β cells. They can present antigens to islet-reactive CD4+ and CD8+ T cells, causing the destruction of islet β cells through cytotoxic T lymphocyte (CTL)-mediated killing, resulting in insufficient insulin release and triggering T1D. This figure was adapted from Nat. Rev. Nephrol. (DOI: 10.1038/nrneph.2017.138) with permission from Springer Nature (License #: 6038731357027).

“**Figures 1** | Mechanisms of B-cell tolerance in healthy individuals. In healthy individuals, non-autoreactive B cells react to foreign antigens through their B-cell receptors (BCRs), successfully migrate to the periphery, and become mature naive B cells. Autoreactive B cells that can bind to self-antigens undergo receptor editing in the bone marrow (a process considered as a central tolerance mechanism). Cells that are successfully edited migrate to the periphery, while those that fail receptor editing are destroyed. Autoreactive B cells with moderate affinity for self-antigens can enter the periphery and acquire tolerance through peripheral tolerance mechanisms, thus preventing activation, proliferation, and losing the ability to produce antibodies against self-antigens. This figure was adapted from Nat. Rev. Nephrol. (DOI: 10.1038/nrneph.2017.138) with permission from Springer Nature (License #: 6038731357027).”

“**Figures 2** | Illustration of B cell tolerance mechanism defects in patients with Type 1 diabetes. In type 1 diabetes, owing to defects in central and peripheral tolerance mechanisms, autoreactive B cells accumulate in the periphery. These cells enter the pancreas orpancreatic lymph nodes to destroy islet β cells. They can present antigens to islet-reactive CD4+ and CD8+ T cells, causing the destruction of islet β cells through cytotoxic T lymphocyte (CTL)-mediated killing, resulting in insufficient insulin release and triggering T1D. This figure was adapted from Nat. Rev. Nephrol. (DOI: 10.1038/nrneph.2017.138) with permission from Springer Nature (License #: 6038731357027).”

The original version of this article has been updated.

